# Variable phenotypic expressivity in a Swiss family with autosomal dominant retinitis pigmentosa due to a T494M mutation in the *PRPF3* gene

**Published:** 2010-03-19

**Authors:** Veronika Vaclavik, Marie-Claire Gaillard, L. Tiab, Daniel F. Schorderet, Francis L. Munier

**Affiliations:** 1 Hopital Ophtalmique Jules Gonin, Lausanne, Switzerland; 2 IRO-Institut de Recherche en Ophtalmologie, Sion, Switzerland, EPFL-Ecole polytechnique fédérale de Lausanne and University of Lausanne, Lausanne, Switzerland

## Abstract

**Purpose:**

To characterize the clinical, psychophysical, and electrophysiological phenotypes in a five-generation Swiss family with dominantly inherited retinitis pigmentosa caused by a T494M mutation in the Precursor mRNA-Processing factor 3 (*PRPF3)* gene, and to relate the phenotype to the underlying genetic mutation.

**Methods:**

Eleven affected patients were ascertained for phenotypic and genotypic characterization. Ophthalmologic evaluations included color vision testing, Goldmann perimetry, and digital fundus photography. Some patients had autofluorescence imaging, Optical Coherence Tomography, and ISCEV-standard full-field electroretinography. All affected patients had genetic testing.

**Results:**

The age of onset of night blindness and the severity of the progression of the disease varied between members of the family. Some patients reported early onset of night blindness at age three, with subsequent severe deterioration of visual acuity, which was 0.4 in the best eye after their fifties. The second group of patients had a later onset of night blindness, in the mid-twenties, with a milder disease progression and a visual acuity of 0.8 at age 70. Fundus autofluorescence imaging and electrophysiological and visual field abnormalities also showed some degree of varying phenotypes. The autofluorescence imaging showed a large high-density ring bilaterally. Myopia (range: −0.75 to −8) was found in 10/11 affected subjects. Fundus findings showed areas of atrophy along the arcades. A T494M change was found in exon 11 of the *PRPF3* gene. The change segregates with the disease in the family.

**Conclusions:**

A mutation in the *PRPF3* gene is rare compared to other genes causing autosomal dominant retinitis pigmentosa (ADRP). Although a T494M change has been reported, the family in our study is the first with variable expressivity. Mutations in the *PRPF3* gene can cause a variable ADRP phenotype, unlike in the previously described Danish, English, and Japanese families. Our report, based on one of the largest affected pedigree, provides a better understanding as to the phenotype/genotype description of ADRP caused by a *PRPF3* mutation.

## Introduction

Retinitis pigmentosa (RP) is a group of inherited retinal degenerations displaying heterogeneity in age of onset, disease progression, and underlying genetic defect. In contrast, the later stages of the disease share common fundus features such as bone spicule pigmentation, chorioretinal atrophy, narrowed vessels, and optic disk pallor [1]. Typical symptoms include night blindness, progressive visual field constriction, and, eventually, legal blindness [2]. Retinitis pigmentosa can result from mutations in more than 45 genes [3]. Transmission is autosomal dominant (ADRP) in about one third of cases, with 10% of these cases caused by mutations in one of three core pre-mRNA splicing factors: Precursor mRNA-Processing factor 3 (*PRPF3*), Precursor mRNA-Processing factor 8 (*PRPF8*), and Precursor mRNA-Processing factor 31 (*PRPF31*). All three genes are involved in the assembly and function of the spliceosome, which clips introns out of the pre-mRNA [4]. In the *PRPF3* gene, two different missense mutations, Thr494Met and Pro493Ser, have been identified in patients with ADRP and simplex cases. Both mutations are clustered within a two-codon stretch in exon 11 and one of the mutations (T494M) is seen repeatedly and has been identified in 1% of patients with ADRP in Japan [5-7]

To date, the genotype/phenotype correlation of T494M in *PRPF3* is limited to a two-generation Japanese family, [7] a seven-generation Danish family, and a four-generation English family, where only a brief description is available [8,9]. Authors [7-9] describe a consistent phenotype: extremely early onset of night blindness, severe visual field constriction, loss of central vision between the fourth and fifth decade, and a flat electroretinogram (ERG) after the age of 30.

In this report, we describe the phenotype of a large five-generation Swiss family from Western Switzerland that is affected with ADRP caused by a missense T494M mutation in *PRPF3*.

## Methods

**Figure 1:**
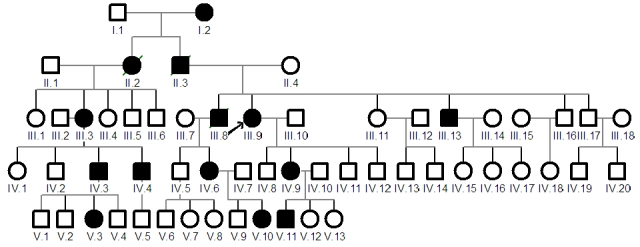
Family tree. The proband is indicated with an arrow. All affected members are indicated by a black symbol.

We studied 11 affected patients and 11 asymptomatic individuals from a family segregating ADRP originating from Western Switzerland (Figure 1). The protocol of the study adhered to the tenets of the Declaration of Helsinki and was approved by the local Ethics Committee DFSP (035.0003–48). After informed consent was obtained, blood samples from affected and unaffected family members were taken on EDTA and DNA was extracted with a Nucleon2 kit as recommended by the supplier (GE Healthcare, Uppsala, Sweden).

All patients had a full history and a complete ophthalmic examination. Medical records were reviewed when accessible. Subjects underwent color fundus photography and autofluorescence imaging (AF). Autofluorescence imaging was obtained using a scanning laser ophthalmoscope (HRA2; Heidelberg Engineering, Heidelberg, Germany) by illuminating the fundus with an argon laser light (488 nm) and viewing the resultant fluorescence through a band-pass filter with a short wavelength cut off at 495 nm [10].

Pupils were dilated using tropicamide 1% and phenylephrine hydrochloride 2.5% before electrophysiology testing. A full-field electroretinographe (ERG) was performed using the International Society for Clinical Electrophysiology of Vision (ISCEV) standards [11] in five patients. The protocol included rod-specific and standard bright-flash ERG, both recorded after a minimum of 20 min dark adaptation. Following 10 min light adaptation, the photopic 30-Hz Flicker cone and transient cone ERG were recorded [11].

Further tests were performed in some patients including multifocal ERG (1 patient/11), Goldmann perimetry (7 patients/11), and optical coherence tomography (OCT; 4 patients/11). Color testing was performed using the Ishihara pseudoisochromatic plates (5 patients/11).

The topographic distribution of autofluorescence across the retina was measured using a gray scale-index of intensity (0–255 units), along the vertical and horizontal meridians, intersecting the foveal pixel [12], and scaled in degrees by assuming a visual angle of 15° between the center of the optic nerve and fovea.

The mutation 1482C>T (Thr494Met) in *PRPF3* exon 11 was identified by microarray screening and confirmed by bidirectional direct sequencing using Big Dye kit 1.1 after amplification of exon 11 by polymerase chain reaction (PCR) with the following primers: 5′-AAG TGA CTT CAA AGA CTG ATT GTT G-3′ and 5′-CTC TTG ATC CAC ACT AGG GTC A-3′.

## Results

### Clinical evaluation

The clinical findings are summarized in Table 1. In all patients, the first clinical symptom present was night blindness. The age of onset varied within family members from early childhood (2 to 7 years) in six cases and to late teens (15 to 20 years) in four cases. One patient did not complain of any night blindness. In family members reporting early onset of night blindness, the disease progression was more severe in terms of visual acuity. In those patients reporting later onset of night blindness, the disease progression was milder, retaining best corrected visual acuity of 0.8 in the better eye until their late seventies. The two younger patients of that group, 39 and 46 years old, both had a best corrected visual acuity (BCVA) of 1.0 and 0.9. Within patients reporting an early nigh blindness, the visual acuity in the better eye was 0.4 in at 53 and 67 years old. In addition, younger patients of that group had reduced vision: 0.4 and 0.3 at 27 years old and 0.6 bilaterally at 45 years old. Except the young four-year-old girl V.3, all patients had myopia (range −0.75 to −8 diopters) and astigmatism. Nystagmus was not present in any patient.

**Table 1:** Summary of clinical findings in affected patients with autosomal dominant retinitis pigmentosa (ADRP) due to T494M mutation in gene PRPF3.

**Patient**	**Sex**	**Age (last visit)**	**Mutation T494M**	**Age of onset night blind**	**VA decimal**	**Refraction**	**Visual field Goldman I/4e**	**Cataract**	**Fundus/AF/ others**
III.3	F	67	+	7 years	OD: 0.3 OS: 0.4	myopia	OD:9° OS:10°	Bilateral cataract operation	Optic nerve palor, migration of pigment in mid periphery
IV.3	M	45	+	2 years	OD: 0.6 OS: 0.6	−8/-2x176° −8/-2x163	OD:7° OS:5°	Subcapsular cataract since 25 years	AF: ring of high density autofluorescence
IV.4	M	27	+	2–3 years	OD: 0.4 OS: 0.3	−4.25/-3x173°-4.75/-3.2x170°		absent	migration of retinal pigment epithelium in mid periphery
V.3	F	4	Not tested	3 years	OD: 0.8 OS: 0.8	hyperopic astigmatism		absent	AF: large hyperfluorescent Rings in both maculas. Pale appearance fundus
III.8	M	53	+	7 years	OD: 0.4 OS: 0.1	myopia		Bilateral cat op aged 43 years	Optic nerve palor
IV.6	F	39	+	15 years	OD: 0.9 OS: 0.8	−7/-3.5x0°-7.75/-2.5x0°	OD:8° OS:10°	Bilateral cat op aged 38 years	Previous buckling for bilateral retinal detach.
V.10	F	10	Not tested	not reported	OD: 0.9 OS: 1.0	−0.5/-1x37°-1.75/-0.5x144°	OD:30° OS:30°	absent	AF: large hyperfluorescent rings in both maculas. Normal appearance fundus
III.9	F	71	+	17–18 years	OD: 0.8 OS: 0.6	−8/-0.5x180°-4/-1.5x105	OD:5° OS:7°	Bilateral cat op aged 60 years	AF: ring of high density autofluorescence
IV.9	F	46	+	15 years	OD: 1.0 OS: 1.0	−5.25/-2.25x74°-4.25/-1x80°	OD:14° OS:12°	absent	AF: ring of high density autofluorescence. No obvious pigment deposits.
V.11	M	22	+	7 years	OD: 1.0 OS: 1.0	−0.75/-0.5x15°+0.75/-1.5x155	OD:30° OS:30°	absent	AF: ring of high density autofluorescence
III.13	M	59	+	20 years	OD: 0.5 OS: 0.8	−7/-2x10°-5.5/-1.5x155		Bilateral cat op aged 49 years	optic neuritis aged 20 years

Abbreviations: NR represents non recordable, AF represents autofluorescence imaging, VA represents visual acuity, OD represents right eye, OS represents left eye.

Fundus appearance varied between patients according to their age and the stage of disease. The youngest subject had normal, although pale appearing fundus. The oldest patients had pale discs, narrowed vessels, and extensive retinal epithelium pigment deposits outside and within the arcades. Macular edema or foveal cyst-like changes were not observed in any patient. In addition, OCT performed in four subjects did not show any intraretinal cysts.

Peripheral visual field constriction with I/4e isopter, as measured by Goldmann kinetic perimetry, varied from 5 to 30 degrees, with an average of 15.3°. In some cases, the testing showed intrafamilial variability: patient III.3 had a visual field of 9° and 10° (right eye and left eye respectively, isopter I4e) when she was 67 years old, while her son IV.3, tested at age 45 years, had a visual field of 7° and 5° (isopter I/4e). A longitudinal data of Goldmann visual field perimetry of subject III.9 over 7 years showed a mild progression from 10° in both eyes when she was 64 years old to 7° and 5° at the last visit.

**Figure 2:**
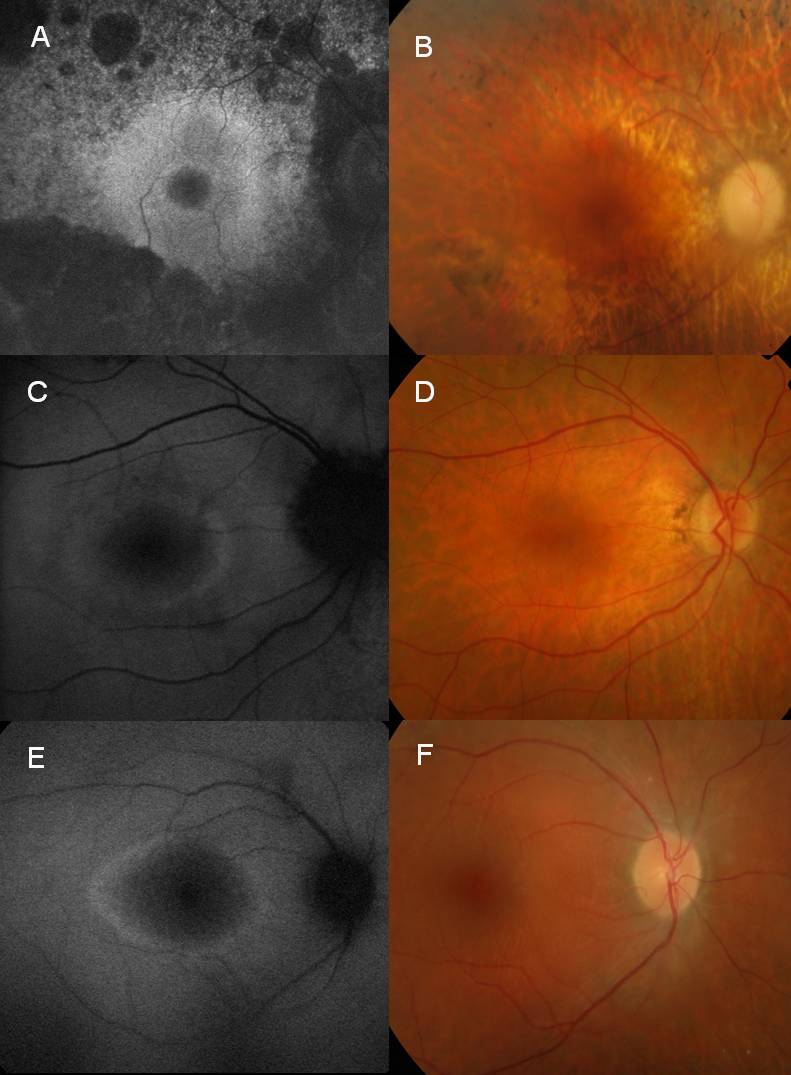
Autofluorescence and fundus photography. This image is showing progressive constriction of perifoveal ring of high density autofluorescence in the grand-mother **A** (Proband III.9), her daughter **C** (IV.9) and the grand son **D** (V.11). **A**: An autofluorescence imaging (AF) image of 70-year-old mother, right eye. Macula with a small hyperfluorescent ring. **B**: Color photo of the same patient. Pale disc, bones spikes at the periphery are shown. **C**: An AF image of the right eye of her daughter, 46-year-old. Macula with a larger hyperfluorescent ring. **D**: Color photo of the same patient. Normal appearance is shown. **E**: An AF image right eye (RE) of 22-year-old grandson. Macula with a larger hyperfluorescent ring. **F**: Color photo of the same patient. Normal appearance is shown.

**Figure 3:**
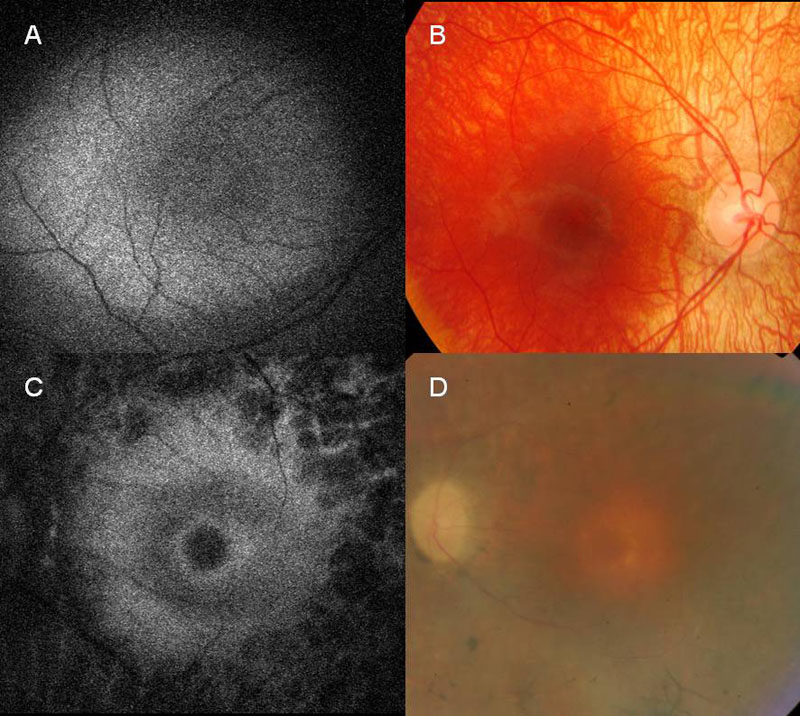
Autofluorescence and fundus photography. This image is showing a ring of high density autofluorescence of a daughter, **A** (V.3) with normal fundus and her father **C** (IV.3). **A**: An autofluorescence imaging (AF) image of four-year-old girl, right eye. Macula with a large hyperfluorescent ring. **B**: Color photo of the same patient. Pale appearance of fundus is shown. **C**: An AF image of her 44-year-old father, left eye. Macula with a small hyperfluorescent ring. **D**: Color photo of the same patient. Pale disc, narrowed retinal vessels, bone spikes at close periphery are shown.

Autofluorescence imaging showed two distinctive patterns of abnormalities. In severely affected cases, there was a decrease or lack of autofluorescence outside or within the arcades (Figure 2A, Figure 3C). All patients who had AF imaging showed a ring of high density autofluorescence (Figure 2A,C,E; Figure 3A,C). The ring of increased AF varied between 4.4° and 26.2° of eccentricity from the fovea. The inner radius of the AF ring in 10 eyes and the outer radius in one patient (V.10) correlated with the Goldmann isopter I/4e average radius (correlation coefficient r^2^: 0.81). In one patient (V.11), the radius of AF ring correlated to a Goldmann I/3e isopter radius.

Figure 2 shows a 70-year-old proband (Figure 2A,B), her 46-year-old daughter (Figure 2 C,D), and 22-year-old grandson (Figure 2E,F). The daughter’s ring of high density AF was 12.4°and 12.6°, while the grandson’s was 15.4° in both eyes.

**Figure 4:**
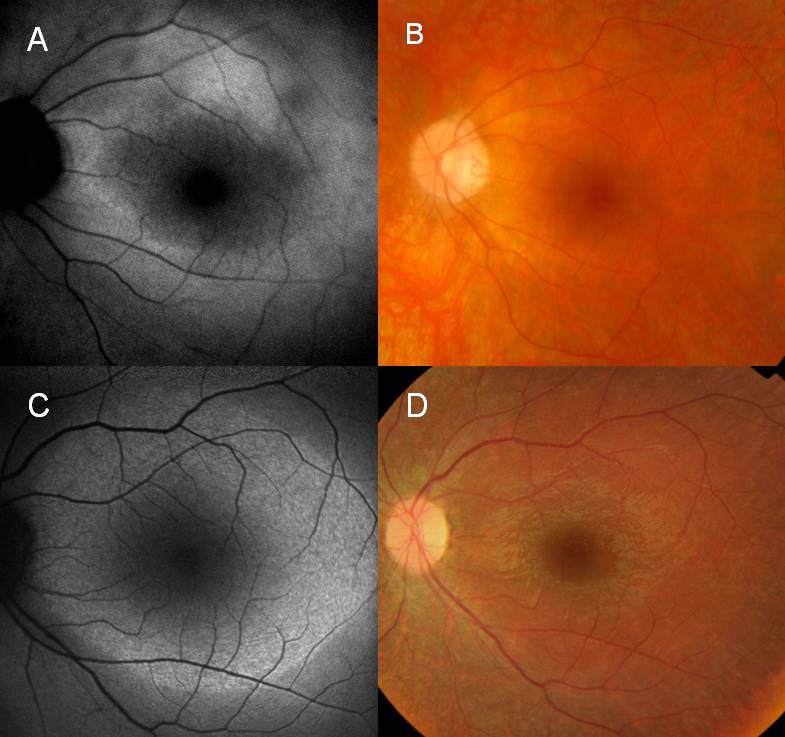
Autofluorescence and fundus photography. This image is showing a ring of high density autofluorescence imaging (AF) of a mother **A** (IV.6) and her asymptomatic daughter **C** (V.10) with normal fundus. **A**: An AF image of 39-year-old mother, left eye. Macula with a hyperfluorescent ring. (**B**) Color photo of the same patient. Slight narrowing of retinal arteries. **C**: An AF image of her 10-year-old asymptomatic daughter, left eye. Macula with a large hyperfluorescent ring **D**: Color photo of the same patient. Normal fundus.

Autofluorescence imaging of the four-year-old girl (Figure 3A) revealed large hyperfluorescent rings in both maculas. She did not complain of any visual problems, but her parents, mostly her affected father, noticed that she was clumsy. A large hyperfluorescent ring on AF imaging was also noticed in a 10-year-old girl who did not report any night blindness (Figure 4C). This area did not correspond to any fundoscopic abnormalities (Figure 4D). The Goldmann visual field testing revealed constriction of isopter I/4e to 30° in both eyes, as well as a dissociation of isopters I/4e and V/4e. Color vision was severely reduced in one patient (III.9) and normal in 3 (IV.3, IV.6, and V.10).

### Functional evaluation

The functional findings are summarized in Table 2. All patients tested had undetectable rod-specific ERG. The photopic ERG showed some intrafamilial variability: a 43-year-old mother (IV.9), who had a late onset of night blindness, had a less affected cone flicker as compared to her 18-year-old son (V.11), with an early onset of nyctalopia. The amplitude (in microvolts) of her photopic 30-Hz flicker ERG was within lower limit of normal (52.9), versus 15.5 in her son. Patient IV.4, 27 at the time of his full field ERG testing, was the most severely affected and had undetectable rod and cone ERGs. His 39-year-old brother (IV.3) was less affected; his photopic ERGs were still detectable, although delayed and reduced.

**Table 2:** Quantitative ERG results, average of both eyes. Amplitudes and implicit times of rod specific, maximal mixed rod-cone responses (max), photopic 30-Hz flicker, and transient photopic tracings are shown.

**Electroretinogram**		**Amplitude (microvolts [mV])**		**Implicit time (ms)**	
		**a-wave**	**b-wave**	**a-wave**	**b-wave**
Rod specific					
Patient 2 IV.3 (39 years)			NR		NR
Patient 3 IV.4 (27 years)			NR		NR
Patient 6 IV.6 (28 years)			NR		NR
Patient 9 IV.9 (46 years)			NR		NR
Patient 10 V.11 (18 years)			NR		NR
**Max ERG**					
Patient 2 (IV.3)	36.5 (167.2*)		69.7 (349.2*)	32.5 (24.3*)	54 (53.5*)
Patient 3 (IV.4)	NR		NR	NR	NR
Patient 6 (IV.6)	43.1 (188.2*)		22 (365.2*)	30.9 (24*)	38 (53.5*)
Patient 9 (IV.9)	90.6 (156.8*)		49.1 (341.2*)	27.5 (24.5*)	52.8 (53.5*)
Patient 10 (V.11)	21.1 (210.3*)		41.5 (382.0*)	20.3 (23.7*)	58 (53.5*)
**Photopic 30-Hz flicker**					
Patient 2 (IV.3)			8.6 (46.4*)		45.3 (31.6*)
Patient 3 (IV.4)			NR		NR
Patient 6 (IV.6)			20.4 (53.4*)		42.9 (31.3*)
Patient 9 (IV.9)			52.9 (42.9*)		41.4 (31.3*)
Patient 10 (V.11)			15.5 (60.8*)		42.4 (31.3*)
**Transient photopic**					
Patient 2 (IV.3)	19.5		38.1 (71*)	19.3	78.3 (32*)
Patient 3 (IV.4)	NR		NR	NR	NR
Patient 6 (IV.6)	8.1		16.4 (81.9*)	22.5	47.0 (32.5*)
Patient 9 (IV.9)	40.4		108.9 (66*)	20.8	39 (32.5*)
Patient 10 (V.11)	13.9		36.4 (91.5*)	18.3	44 (32.5*)

Asterisk (*) indicates the lower threshold of normal amplitude (age-adjusted) or implicit time, defined as two standard deviations below the mean of normal controls. NR represents non recordable.

Multifocal ERGs of patient III.9 showed a widespread reduction with no preservation of the response associated with the central stimulus element.

### Mutation detection

**Figure 5:**
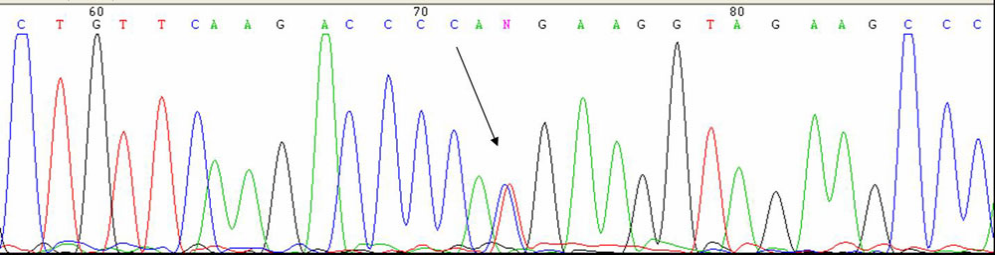
Electropherogram. This image is showing mutation 1482 C>T (Thr494Met) in Precursor mRNA-Processing factor 3 (PRPF3). The arrow showing the “double hill” at position 73, corresponding to heterozygote mutation.

Using microarray screening (AsperOphthalmics, Tartu, Estonia), the proband (patient III.9) was found to have a Thr494Met mutation in the *PRPF3* gene. Presence of the c.1482C>T transition was confirmed using direct bidirectional sequencing of exon 11 (Figure 5). No previously reported mutation was detected in the *CA4*, *FSCN2*, *IMPDH1*, *NRL*, *PRPF31*, *PRPF8*, *RDS*, *RHO*, *ROM1*, *RP1*, *RP9*, *CRX*, *TOPORS*, and *PNR* genes.

The identified mutation co-segregated with the disease in all affected family members tested (9 patients/11) and it was not observed in 96 control individuals.

## Discussion

In this report we describe the phenotype-genotype correlation in a five-generation Swiss family with a Thr494Met mutation in *PRPF3*. This is one of the largest families described with this mutation and is the first evidence for variable intrafamilial expressivity.

Only two missense mutations have been described in *PRPF3*, Thr494Met and Pro493Ser, as reported by Chakarova [13]. Both codons 494 and 493, placed in the C-terminal region of the protein, are highly conserved and this region has been suggested to interact with other proteins [13,14]. The T494M mutation is considered to be the most common mutation, as it has been reported to segregate in several families from various ethnic background (English, Danish, Spanish, Japanese, and North American), with a frequency of 1% among patients with ADRP.

The previous T494M-associated phenotype was consistent and described an onset of night blindness in early infancy, a visual field loss, and decreased visual acuity between the ages of 30 and 40, as well as a flat ERG after the age of 30 [7-9]. Only one patient carrying a mutation in *PRPF3* has been examined in the Spanish family [5] and was described as having a mild phenotype with first symptoms (night blindness) at the age of 40.

Our Swiss family presented with variable expressivity in regards to the age of onset of night blindness and disease progression, as shown by a variable deterioration of central visual acuity. The first group of patients reported nyctalopia at a very early age, with subsequent early and severe deterioration of central vision. The BCVA of 0.4 was reported after their fifties and was also measured in younger patients, confirming a severe disease progression. The second group reported night blindness in their late teens, early twenties, and all patients from that group retained good visual acuity, even after the age of 70 (BCVA: 0.8). Only the first group’s phenotype correlates with the phenotype of the other families described [7-9]. All patients had some degree of myopia and astigmatism, which has also been reported in some members of the Danish family [9] and in one of the three patients described in the Japanese family [7].

Goldmann kinetic perimetry visual field testing also showed some degree of intrafamilial variability, as a younger patient (IV.3) was more severely affected then his mother and had the same degree of visual field constriction as a 71-year-old subject (III.9) from that family. In addition, photopic ERGs also showed some intrafamilial variability, as some younger patients were more affected then older ones.

The autofluorescence imaging revealed a parafoveal ring of high density autofluorescence in all tested patients. An abnormal ring of hyperautofluorescence, which is not visible on slit-lamp biomicroscopy, is frequently observed and has been reported in ADRP, autosomal recessive retinitis pigmentosa (arRP), and X-linked RP [15-17]. It has also been documented in other retinal dystrophies, including Leber congenital amaurosis [18], cone-rod dystrophy [19], Best disease [20], and X-linked retinoschisis [21]. The hyperfluorescent ring might be due to early cellular dysfunction and the overproduction of lipofuscin [22]. It is a non-specific manifestation, but testing by pattern and multifocal electroretinography, OCT, and microperimetry revealed that the retinal function inside the ring was more preserved than outside the ring [10,15,16,23,24]. Our results confirm that the size of the AF ring correlates well with Goldmann perimetry, as has been previously reported [10,16,24,25]. In our study, the average diameter of the AF ring correlated with isopter I/4e, as opposed to isopter II/4e as described in a previous study [24], or isopter V/4 in another study [25]. This difference might be due to variability and a suprathreshold stimulus of kinetic perimetry described in patients with RP [26].

Autofluorescence is useful to confirm a retinal dystrophy and to monitor its progression. In this family, a 10-year-old girl claimed to be asymptomatic and her clinical examination was unremarkable (Figure 4D). The diagnosis of the disease was only suspected based on family history and visual field, and was confirmed with AF imaging: she had bilateral parafoveal large rings of high density AF (Figure 4C) [27]. It might often be challenging to obtain reliable ERG results in small children with retinal dystrophies, partly because the normative values of healthy children are not easily available in most diagnostic centers. Our study suggests that AF imaging can be a more child-friendly tool in diagnosing RP in young or asymptomatic children.

In summary, our report describes a spectrum of phenotypes of ADRP due to a *PRPF3* T494M mutation in a large five-generation family, in which we examined 11 affected members. Our series also highlights the importance of using AF in evaluating young children and in monitoring adult patients.

## References

[1] Heckenlively JR Yoser SL Friedman LH Oversier JJ Clinical findings and common symptoms in retinitis pigmentosa. Am J Ophthalmol 1988 105 504 11 3259404 10.1016/0002-9394(88)90242-5

[2] Daiger SP Bowne SJ Sullivan LS Perspective on genes and mutations causing retinitis pigmentosa. Arch Ophthalmol 2007 125 151 8 17296890 10.1001/archopht.125.2.151 PMC2580741

[3] Hartong DT Berson EL Dryja TP Retinitis pigmentosa. Lancet 2006 368 1795 809 17113430 10.1016/S0140-6736(06)69740-7

[4] Smith DJ Query CC Konarska MM “Nought may endure but mutability”: spliceosome dynamics and the regulation of splicing. Mol Cell 2008 30 657 66 18570869 10.1016/j.molcel.2008.04.013 PMC2610350

[5] Martinez-Gimeno M Gamundi MJ Hernan I Maseras M Milla E Ayuso C Garcia-Sandoval B Beneyto M Vilela C Baiget M Antinolo G Carballo M Mutations in the pre-mRNA splicing-factor genes PRPF3, PRPF8, and PRPF31 in Spanish families with autosomal dominant retinitis pigmentosa. Invest Ophthalmol Vis Sci 2003 44 2171 7 12714658 10.1167/iovs.02-0871

[6] Sullivan LS Bowne SJ Birch DG Hughbanks-Wheaton D Heckenlively JR Lewis RA Garcia CA Ruiz RS Blanton SH Northrup H Gire AI Seaman R Duzkale H Spellicy CJ Zhu J Shankar SP Daiger SP Prevalence of disease-causing mutations in families with autosomal dominant retinitis pigmentosa: a screen of known genes in 200 families. Invest Ophthalmol Vis Sci 2006 47 3052 64 16799052 10.1167/iovs.05-1443 PMC2585061

[7] Wada Y Itabashi T Sato H Tamai M Clinical features of a Japanese family with autosomal dominant retinitis pigmentosa associated with a Thr494Met mutation in the HPRP3 gene. Graefes Arch Clin Exp Ophthalmol 2004 242 956 61 15085354 10.1007/s00417-004-0923-x

[8] Inglehearn CF Tarttelin EE Keen TJ Bhattacharya SS Moore AT Taylor R Bird AC A new dominant retinitis pigmentosa family mapping to the RP18 locus on chromosome 1q11–21. J Med Genet 1998 35 788 9 9733043 10.1136/jmg.35.9.788 PMC1051440

[9] Xu SY Schwartz M Rosenberg T Gal A A ninth locus (RP18) for autosomal dominant retinitis pigmentosa maps in the pericentromeric region of chromosome 1. Hum Mol Genet 1996 5 1193 7 8842740 10.1093/hmg/5.8.1193

[10] Robson AG Saihan Z Jenkins SA Fitzke FW Bird AC Webster AR Holder GE Functional characterisation and serial imaging of abnormal fundus autofluorescence in patients with retinitis pigmentosa and normal visual acuity. Br J Ophthalmol 2006 90 472 9 16547330 10.1136/bjo.2005.082487 PMC1856999

[11] Marmor MF Hood DC Keating D Kondo M Seeliger MW Miyake Y Guidelines for basic multifocal electroretinography (mfERG). Doc Ophthalmol 2003 106 105 15 12678274 10.1023/a:1022591317907

[12] Lois N Halfyard AS Bird AC Fitzke FW Quantitative evaluation of fundus autofluorescence imaged “in vivo” in eyes with retinal disease. Br J Ophthalmol 2000 84 741 5 10873986 10.1136/bjo.84.7.741 PMC1723525

[13] Chakarova CF Hims MM Bolz H bu-Safieh L, Patel RJ, Papaioannou MG, Inglehearn CF, Keen TJ, Willis C, Moore AT, Rosenberg T, Webster AR, Bird AC, Gal A, Hunt D, Vithana EN, Bhattacharya SS. Mutations in HPRP3, a third member of pre-mRNA splicing factor genes, implicated in autosomal dominant retinitis pigmentosa. Hum Mol Genet 2002 11 87 92 11773002 10.1093/hmg/11.1.87

[14] Wang A Forman-Kay J Luo Y Luo M Chow YH Plumb J Friesen JD Tsui LC Heng HH Woolford JL Jr Hu J Identification and characterization of human genes encoding Hprp3p and Hprp4p, interacting components of the spliceosome. Hum Mol Genet 1997 6 2117 26 9328476 10.1093/hmg/6.12.2117

[15] Robson AG El-Amir A Bailey C Egan CA Fitzke FW Webster AR Bird AC Holder GE Pattern ERG correlates of abnormal fundus autofluorescence in patients with retinitis pigmentosa and normal visual acuity. Invest Ophthalmol Vis Sci 2003 44 3544 50 12882805 10.1167/iovs.02-1278

[16] Robson AG Michaelides M Saihan Z Bird AC Webster AR Moore AT Fitzke FW Holder GE Functional characteristics of patients with retinal dystrophy that manifest abnormal parafoveal annuli of high density fundus autofluorescence; a review and update. Doc Ophthalmol 2008 116 79 89 17985165 10.1007/s10633-007-9087-4 PMC2244701

[17] Wegscheider E Preising MN Lorenz B Fundus autofluorescence in carriers of X-linked recessive retinitis pigmentosa associated with mutations in RPGR, and correlation with electrophysiological and psychophysical data. Graefes Arch Clin Exp Ophthalmol 2004 242 501 11 15173948 10.1007/s00417-004-0891-1

[18] Scholl HP Chong NH Robson AG Holder GE Moore AT Bird AC Fundus autofluorescence in patients with leber congenital amaurosis. Invest Ophthalmol Vis Sci 2004 45 2747 52 15277500 10.1167/iovs.03-1208

[19] Downes SM Payne AM Kelsell RE Fitzke FW Holder GE Hunt DM Moore AT Bird AC Autosomal dominant cone-rod dystrophy with mutations in the guanylate cyclase 2D gene encoding retinal guanylate cyclase-1. Arch Ophthalmol 2001 119 1667 73 11709018 10.1001/archopht.119.11.1667

[20] Jarc-Vidmar M Kraut A Hawlina M Fundus autofluorescence imaging in Best's vitelliform dystrophy. Klin Monatsbl Augenheilkd 2003 220 861 7 14704944 10.1055/s-2003-812555

[21] Tsang SH Vaclavik V Bird AC Robson AG Holder GE Novel phenotypic and genotypic findings in X-linked retinoschisis. Arch Ophthalmol 2007 125 259 67 17296904 10.1001/archopht.125.2.259 PMC2757628

[22] Fleckenstein M Charbel IP Helb HM Schmitz-Valckenberg S Scholl HP Holz FG Correlation of lines of increased autofluorescence in macular dystrophy and pigmented paravenous retinochoroidal atrophy by optical coherence tomography. Arch Ophthalmol 2008 126 1461 3 18852430 10.1001/archopht.126.10.1461

[23] Lima LH Cella W Greenstein VC Wang NK Busuioc M Smith RT Yannuzzi LA Tsang SH Structural assessment of hyperautofluorescent ring in patients with retinitis pigmentosa. Retina 2009 29 1025 31 19584660 10.1097/IAE.0b013e3181ac2418 PMC2749567

[24] Popovic P Jarc-Vidmar M Hawlina M Abnormal fundus autofluorescence in relation to retinal function in patients with retinitis pigmentosa. Graefes Arch Clin Exp Ophthalmol 2005 243 1018 27 15906064 10.1007/s00417-005-1186-x

[25] Murakami T Akimoto M Ooto S Suzuki T Ikeda H Kawagoe N Takahashi M Yoshimura N Association between abnormal autofluorescence and photoreceptor disorganization in retinitis pigmentosa. Am J Ophthalmol 2008 145 687 94 18242574 10.1016/j.ajo.2007.11.018

[26] Ross DF Fishman GA Gilbert LD Anderson RJ Variability of visual field measurements in normal subjects and patients with retinitis pigmentosa. Arch Ophthalmol 1984 102 1004 10 6743076 10.1001/archopht.1984.01040030806021

[27] Wabbels B Preising MN Kretschmann U Demmler A Lorenz B Genotype-phenotype correlation and longitudinal course in ten families with Best vitelliform macular dystrophy. Graefes Arch Clin Exp Ophthalmol 2006 244 1453 66 16612637 10.1007/s00417-006-0286-6

